# Hepatic Abscess Opening Externally

**Published:** 1837

**Authors:** G. C. Starbuck

**Affiliations:** Rochester, Ohio


ART. II.—HEPATIC ABSCESS OPENING EXTERNALLY. DISCHARGE OF BILIARY CALCULI. RECOVERY. Communicated by Dr. G. C. Starbuck, of Rochester, Ohio.

Mrs. L. of Rochester, aged about 53, an emigrant from Virginia, who had previously enjoyed good health, was attacked, in October 1835, with remitting fever, attended with pulmonic and hepatic congestion. An active antiphlogistic treatment soon arrested the febrile action; but it was succeeded by an induration of the right lobe of the liver, and disordered functions of the stomach. By the aid of blue mass, tonics, and counter-irritation, her general health improved. She remained subject, however, to occasional exascerbations of pain in the right hypochondriac and epigastric regions, attended with nausea and vomiting, diminished arterial action, and great prostration of strength. These exascerbations continued to increase both in frequency and severity, for the ensuing 12 months; sometimes preceded by rigors and an increase of the hepatic tumors, and succeeded by purulent evacuations per annum.

About this time the tumor (having descended nearly to the pelvis) pointed externally about 1 1-2 inches from the superior anterior spinous process of the ilium, nearly on a line from that process to the ensiform cartilage. The integuments gave way, so as to admit the discharge of a thin sanious matter, in small quantities. After a continuance of this discharge, for two or three weeks, without affording any relief to the patient, the opening closed.

This was the condition of the parts on the first of December 1836, when I was called into consultation with Dr. Thacker, who had had charge of the case for some months. The patient was considerably emaciated. The functions of digestion and assimilation much impaired, and she scarcely passed a week without having an exascerbation of pain and purulent discharges. Fluctuation was tolerably distinct in the tumor, and the integuments, around where the fistula had been, for the diameter of an inch, were discolored and protruded.

After a patient investigation, we agreed to adopt the hint afforded us by the *vis medicatrix naturæ.* Not knowing the extent of the adhesion, we concluded to introduce the lancet where the abscess had been discharging, with the point directed towards the crura of the diaphragm, being confident that we could, in that manner, reach the collection of pus, with a common abscess lancet. But the result somewhat disappointed us.

The patient was placed in an erect position, and I secured the tumor as well as I could, by grasping it with my left hand, and introduced my lancet. I found much more resistance in the parts to be divided than I anticipated, having to make two or three thrusts, and elevate the point each time, before I could carry it to the depth of 1 1-2 inches. No hemorrhage succeeded the operation, and but trifling discharge of pus. We concluded, however, that by keeping the orifice open the contents of the abscess would shortly be discharged; wherefore we introduced a tent, and applied an emolient poultice, and the ensuing morning the tent was forced out of the wound, and the surface of the poultice covered with biliary calculi.

They were about the size of garden peas, of a pale orange color, and about the consistence of yellow wax, only not so tenacious, nearly equal in size, of a spherical form, but exhibiting some flattened surfaces, such as would be produced by crowding them together, and in number about fifteen or twenty.

Our patient would not submit to an examination with the probe, but there was no subsequent discharge of calculi, and her health rapidly improved. The orifice discharged but little, yet the tumor gradually subsided, and at the end of three months from the operation, it had nearly disappeared, and the integuments were sound.

The foregoing case affords a beautiful illustration of the efforts of nature to throw off a morbid excitant, and of the failure of those efforts, until they were assisted by art.

At different times, she had nearly all the symptoms characteristic of the passage of calculi through the biliary ducts; and the purulent discharges from the rectum must have been occasioned, by the hepatic abscess bursting into some part of the intestines. Although none of the calculi were discovered in the evacuations, yet the probability is, that some escaped in both of those ways, and were either unnoticed, or dissolved in the contents of the bowels. But these openings not being sufficient for their complete evacuation, an attempt was made to discharge them through the parieties of the abdomen. The natural opening thus formed, being still insufficient for their escape, I know not what expedient would have been her next resort. But here Art "came to the rescue," and by making a sufficient opening at the point where Nature had made an unsuccessful attempt, removed at once the cause of all the phenomena, and the health was speedily restored.

P. S. Since writing the above, I called to see the patient, and found her engaged in domestic affairs, with a cheerful, healthy countenance, and considerable muscular strength. She informs me, however, that the orifice has never remained long closed. There now appears to be a small fistulous opening, the discharge from which is very trifling, and a little induration extending from it to the extremity of the lower rib; and she has had no pain or soreness of the parts, since the discharge of the calculi.

April, 1837.

